# Extending the classification of healthcare-associated bloodstream infection to other main foci: respiratory, urinary and intra-abdominal

**DOI:** 10.1186/cc9634

**Published:** 2011-03-11

**Authors:** C Cardoso, O Ribeiro, I Aragão, A Costa-Pereira, A Sarmento

**Affiliations:** 1 Hospital de Santo António, Porto, Portugal; 2 Faculty of Medicine, University of Porto, Portugal; 3 Hospital de São João, Porto, Portugal

## Introduction

Healthcare-associated infection (HCAI) is a growing phenomena associated with the increase of the outpatient clinical care. Friedman in 2002 proposed a new classification for healthcare-associated bloodstream infections, suggesting that they are different from nosocomial and community-acquired infections [1]. The authors extend this classification to other main focus of infection: respiratory, urinary and intra-abdominal.

## Methods

A prospective cohort study (1 year), in five wards of a university hospital, including all consecutive adult patients that met the CDC definition of infection. Only the first episode of infection was characterized. They were classified in community-acquired (CAI), HCAI (using Friedman's classification [1]) and hospital-acquired (HAI), and data on the host and the infectious episode were collected.

## Results

**Figure 1:**
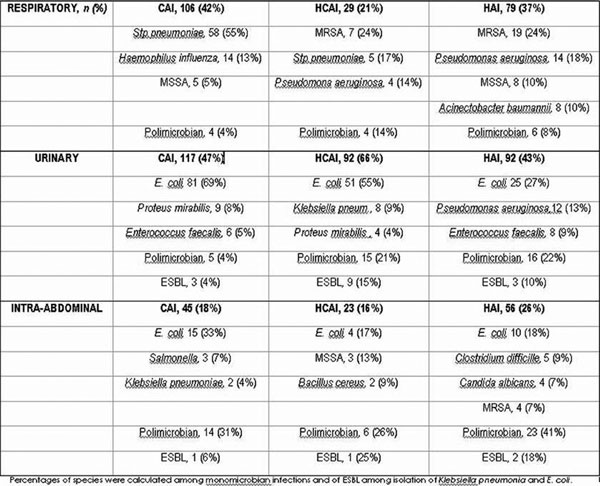
**Microbiological profile according to the focus of infection**.

See Figure 1. We included 1,035 patients: 493 (48%) with CAI, 225 (22%) with HCAI and 317 (31%) with HAI.

## Conclusions

Differences were observed according to the type and focus of infection. These results reinforce the need for this classification and probably the need for specific antibiotic therapy guidelines for this group of patients.
